# 

**Published:** 1842-08-13

**Authors:** 


### CONTENTS OF No. 33.

Dr. Jno. T. Sharpless's Case of Preservation of the Human Body by the Acetate of Alumina, - 513
Dr. Elkinton's Case of Apoplexy, - 514
Dr. M. W. Wilson's Clinical Reports:
Case of Dropsy dependant upon Cirrhosis of the Liver, 515
ANALECTA: On the Evolution of Light from the Living Human Subject, 517
Remarks by Dr. R. Coates, - 521
Disease of the Tarsal Bones—Chopart's Operation, - 522
Dr. Martin Barry on Fibre, - 523
Biographical Sketch of the late Sir Charles Bell, - 524
Dr. Winn on the occasional tendency of Iodine to produce Inflammation of the Joints, - 527
Test for Arsenic, - 528

